# Looking into the Test Tube: The Birth of IVF on British Television

**DOI:** 10.1017/mdh.2019.6

**Published:** 2019-04

**Authors:** Katharine Dow

**Affiliations:** Senior Research Associate, Reproductive Sociology Research Group (ReproSoc), University of Cambridge, 16 Mill Lane, Cambridge, CB2 1SB, UK

**Keywords:** IVF, Louise Brown, Television, Science communication, Britain, 1970s

## Abstract

The birth of Louise Brown, the world’s first ‘test-tube baby’, has come to signify the moment at which technologically assisted human reproduction became a re ality. This was a highly mediated and visible reality, as this article explores through the example of a British television documentary about Louise Brown broadcast when she was just six weeks old, ‘To Mrs Brown… A Daughter’ (Thames Television, 1978). In the article, I discuss the programme alongside data from an interview with its producer, Peter Williams. Williams sought to convince the public that IVF was morally acceptable and to cultivate sympathy for the infertile through this film. I will consider how he went about this by focusing on the programme’s visual presentation of Louise Brown, Peter Williams’ aims in making the film and his sympathetic relationship with the ‘pioneers’ of IVF, gynaecologist Patrick Steptoe and physiologist Robert Edwards. I will conclude with a discussion of the political implications of this film and how it contributed to the normalisation of IVF at a pivotal moment in its history.

The birth of the world’s first ‘test-tube baby’, Louise Brown, on 25 July 1978 in Oldham, northwest England has come to represent the origin story of technologically assisted human reproduction. At 8pm on 7 September 1978, when Louise was just six weeks old, ITV – at the time, the only commercial television channel in the UK – screened a documentary about her called ‘To Mrs Brown… A Daughter’. It told the story of Louise’s conception and birth from the perspective of the main characters involved – her parents, Lesley and John Brown, and the ‘pioneers’ of in vitro fertilisation (IVF), Patrick Steptoe and Robert Edwards. Steptoe (1913–88) was a practising consultant obstetrician, trained at St George’s Hospital Medical School, who had been working at Oldham General Hospital since 1951. He became known to Edwards for his pioneering work in laparoscopy, which he developed in his practice in Oldham. Edwards (1925–2013) received his PhD from the University of Edinburgh in 1955 and, after several research positions, in 1963 he went to the University of Cambridge as a Ford Foundation Research Fellow. He became a reader in physiology at Cambridge in 1969 and remained at Cambridge until retirement, receiving the Nobel Prize in Physiology or Medicine for his work on the development of IVF in 2010.

‘To Mrs Brown’ launched the weekly Thames Television documentary series *This Week* after it was renamed *TV Eye*. The series, which focused on current affairs of all kinds and was hosted by a number of well-known presenters, started in 1956 and ran until 1992 (reverting to its original name in 1986). This episode was produced and presented by Peter Williams, now in his 80s, who has had an extensive career in production and broadcasting at both independent television companies and the BBC since the 1960s. It was to be the first of three programmes about IVF that he would make in the space of four years for British and American television. As well as analysing the content of this particular programme, in this article I will draw on an interview that I conducted with Peter Williams about the programme in March 2015 and on answers he gave at a Q&A during a conference organised as part of the IVF Histories and Cultures Project at the University of Cambridge in December 2014.^1^

As the editors of this special issue note in their introduction, reproductive politics is not only about governments’ regulation of sexuality, marriage or child rearing but also how reproductive knowledge is communicated and circulated. In this article, I will examine how ‘To Mrs Brown… A Daughter’ contributed to the representations of IVF that were available to the public in 1978 and what part its producer, his aims in making the programme and his relationship to Patrick Steptoe and Robert Edwards played in its form and content. My analysis is driven by questions about how stories are told, who gets to tell them and how certain stories are deemed worth telling (and others not). As Stuart Hall argued, media representations do not simply re-present events to viewers as they are: they are constitutive of them. IVF was in the late 1970s a contentious new technology that seemed to intervene in the most intimate bodily functions and private relationships. It suggested the necessity for a whole new vista of legal regulation, but, for many commentators, it also had the potential to challenge and remould normative conceptions of reproduction and kinship, at a time in which social conservatism was on the rise and ‘family values’ had great currency in the UK.

1978 marked a point of transition in which, broadly speaking, the progressive politics of the 1960s were giving way to disquiet about trade unions’ power, racial tensions and concerns about the demise of institutions like the nuclear family. The popular appeal of traditionalism and neoliberalism at this time was reflected in the election of the Conservative Party with Margaret Thatcher as prime minister in 1979. Like all assisted reproductive technologies (ART), IVF can be put to both radical and normative ends. It has always had the capacity to be not simply a way of helping infertile married couples have biogenetically related children but of opening up new forms of family formation, increasing the timespan in which women can become pregnant and creating the potential to select embryos once they have been conceived.^2^

Given the socio-historical context of 1978, the way in which the story of Louise Brown’s conception and birth was told, and whether this chimed with viewers and public commentators, was likely to have important consequences for the acceptance of both her and the technology with which she was conceived. Unfortunately, I do not have the data to discuss viewers’ reception of this film, but given its prime-time slot (bookended by favourites *George and Mildred* and *The Sweeney*), it is likely to have had a healthy audience. Some examples of the critical response to the programme are available in the newspaper archives. The *Daily Mail*, which had exclusive coverage rights to the Browns’ story in the press, offered a positive preview of the programme that emphasised its ‘exclusive’ status, noted the paper’s collaboration with the filmmakers and described Peter Williams as having ‘reason to be proud of his share in the film’. The programme was shown in the United States the following year on WNET-TV and the *New York Times* gave it a glowing review, describing the Browns, Steptoe and Edwards as ‘attractive’, dismissing the moral controversy surrounding the birth as baseless and concluding that the programme ‘is a model of straightforward, unpretentious, civilized documentary-making’. Nancy Banks-Smith in *The Guardian* offered some mild criticism, which was related to the documentary’s style rather than any opprobrium about the subject matter: ‘Like Louise Brown, the programme was remarkable only in being the first of its kind. A nice, clear, uncritical job of work with any reservations implicit, not explicit.’ While she might have found the filmmaking to be a little pedestrian, Banks-Smith enthusiastically described ‘the TV pictures through a medical telescope into the womb [i.e. laparoscope]’ as ‘new and fascinating’; I will discuss this laparoscopy footage later in this article.^3^

The birth of Louise Brown was a worldwide media event, and stories of IVF and other ART have continued to feature prominently in news media, science documentaries, fiction and the arts ever since. But despite the intensity of the media coverage and the worldwide controversy, I have found from analysing contemporary newspaper articles that the dominant narrative in the British news media in 1978 was a positive story of IVF being the answer to the scourge of infertility and a way of helping ‘ordinary’ married couples have babies. While this was a time in which overpopulation discourse was influential in both public debate and funding for medical research, news journalists took it for granted that infertility was a serious and tragic condition, especially for women, and so the trope of the ‘desperate’ infertile woman or couple was in common circulation, and many newspapers greeted the news of Lesley Brown’s pregnancy as representing ‘hope’ for the infertile.^4^

The dominant narrative in the British press normalised IVF by placing it within the bounds of ‘respectable’ heterosexual family formation. ‘To Mrs Brown’ was similarly sympathetic to IVF and was driven, as I shall discuss, by Peter Williams’ desire to help the public understand and accept IVF. After providing a précis I will go on to analyse three aspects of the programme. First, I will describe how Louise’s status as a normal baby was established through visual verification in the programme. I will then go on to discuss two other interrelated aspects of the film: Peter Williams’ role and aims in making the programme and the relationship between Williams, Patrick Steptoe and (to a less prominent extent) Robert Edwards. Finally, I will discuss the politics of representation of IVF in this case, arguing that, with the aid and encouragement of Steptoe and Edwards, in this programme, Williams was able to harness the visual cultures of both television and IVF to make IVF appear everyday, safe and acceptable because it appeared to be simply a means of helping infertile couples have babies rather than a challenge to the moral or natural order.

As Sarah Franklin notes, we need to draw attention to the precepts behind stories of scientific innovations, including assumptions about science being a harbinger of progress and scientific ‘discoveries’ as detective stories driven by a thirst for truth, while also bearing in mind Raymond Williams’ caution about technological determinism in how we think about television in particular. As Hall argued, representations arise out of shared meanings and understandings – audiences will accept those narratives that seem to ring true, after all. In the case of Louise Brown, common understandings about the meanings of babies – especially blonde, blue-eyed healthy ones with loving, married, white parents – played a crucial role in constituting the meaning of IVF and thereby its political, legal and ethical status.^5^ Peter Williams told me in our interview that he wanted to help the public understand, and thereby come to accept, IVF through making this film. In this article, I will examine some of the ways in which he tried to realise this aim through this programme and the normative assumptions that informed his approach.^6^

## 1 ‘To Mrs Brown…’: A Summary

The programme opens with a wide aerial shot of the town of Oldham. The air is slightly hazy. Industrial chimneys and terraced houses are the most prominent features in the picture, though rolling green hills are just visible in the background. Peter Williams has a resonant voice with a Received Pronunciation accent. He provides a voiceover throughout the film, which opens with, ‘At thirteen minutes to midnight on July twenty-fifth 1978, in the Lancashire cotton-mill town of Oldham, there was born in the local hospital to an English couple, Mr and Mrs John Brown, a baby daughter.’ The camera pans right and zooms in on what the voiceover implies is Oldham General Hospital. After focusing on the hospital for a few seconds, the shot dissolves into an image of a blonde, blue-eyed baby lying on a mat on a carpet, waving her arms, and the title of the programme appears above her. Williams has explained that the title, ‘To Mrs Brown… A Daughter’, was inspired by the notices put in local newspapers announcing the birth of a child.^7^

**Figure 1:**
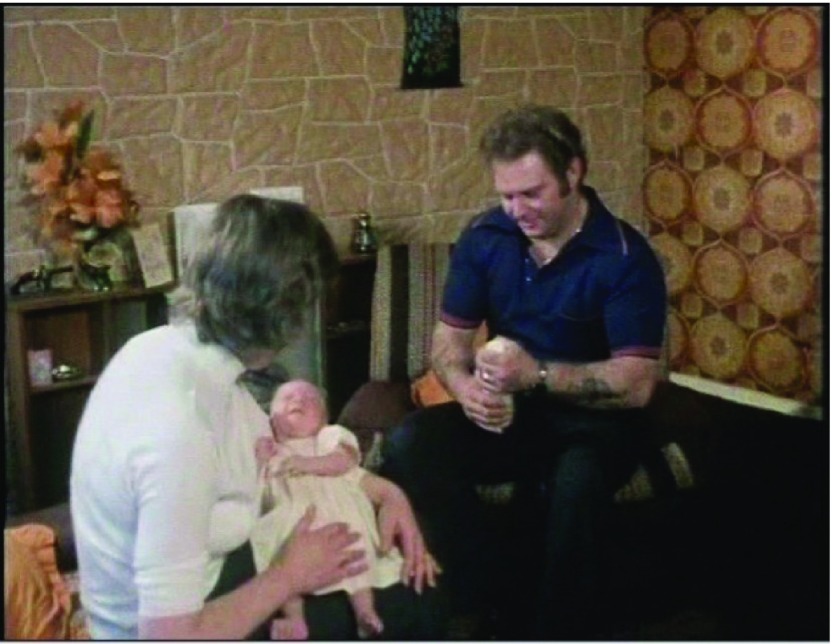
A ‘refreshingly ordinary’ family – Lesley, Louise and John Brown at home in ‘To Mrs Brown… A Daughter’.

The film then cuts to a shot of the interior of the Browns’ house, with John Brown walking into view and down the hallway into the living room. He is holding a baby’s milk bottle and walks with a slight swagger. The camera follows him as he enters the living room, which is decorated in keeping with the exuberant taste of the time. He sits down opposite Lesley. She is sitting on a sofa, holding baby Louise, who is crying. As he hands the bottle to Lesley, his strong Bristolian accent and the tattoos on his arms and hands are noticeable. Lesley is first viewed from the side. Her pose is a quintessentially maternal one, which might bring to mind the Virgin Mary for some but also reflects the modesty that many journalists described as a quintessential feature of Lesley’s personality (see Figure 1).

The remainder of the film, which is fifty minutes long, is composed of several main elements. The most substantial of these is excerpts from Peter Williams’ interviews with Lesley and John Brown and with Patrick Steptoe at their respective homes. Steptoe and Edwards each appear on their own. John and Lesley Brown are shown separately and together, though it is quite possible that the extracts shown in the film were all filmed with husband and wife both in the room. Alongside these interviews, contextualising scenes give a sense of these main characters in their everyday lives. So, Steptoe is filmed listening to cricket commentary on the car radio while driving his white Mercedes through Oldham, Edwards is seen walking in the Yorkshire countryside with his five daughters, the Browns are shown taking Louise for a walk in a park near the Clifton Suspension Bridge and we see Lesley bathing Louise and putting her in her cot.

The everyday, domestic nature of these scenes is notable. As I will describe later, some of the programme’s pivotal ‘action’ scenes take place in laboratory and clinical settings, yet these interview sections, in which the protagonists are given the space to articulate their own views on and experiences of IVF, occur in domestic settings. This lends a relatable everydayness to the story as well as humanising Steptoe and Edwards – ‘mad’ scientists are after all typically depicted as tied to their labs and divorced from normal life – and allowing the Browns to appear as an ordinary, loving couple whose main interest is building and maintaining their family life.^8^

In 1978, television was a quintessentially domestic technology and typically viewed in a family setting. This is not only relevant in terms of the likely context in which viewers would have watched ‘To Mrs Brown’ but, as Roger Silverstone pointed out, is also relevant to the politics of representation on television. Writing in 1994, Silverstone argued that television’s power comes from its embeddedness in everyday and family life, so that, ‘We can no more think of television as anything other than a necessary component of that domesticity than we can think of our domesticity without seeing both in the machine and the screen a reflection and an expression of that domestic life.’ Silverstone pointed out that this reflects a bourgeois reordering of life into public and private, professional and domestic realms; this worldview was also prominent in the Thatcherite political rhetoric of the late 1970s.^9^

As well as these visual sketches, each of the characters is described in a pithy shorthand in the voiceover, so the Browns are ‘a refreshingly ordinary couple’, Steptoe is a ‘man of style’ and Edwards is both ‘Edwards the scientist’ and ‘Edwards the countryman’. The other major element of the film is Williams’ documentation of Steptoe and Edwards’ clinical work, which is dominated by footage of Steptoe performing laparoscopy. The programme also includes some scenes from the government’s Central Office of Information film of Louise Brown’s birth by caesarean section (see below) and a series of short interviews with other couples who were seeking IVF treatment with Steptoe at the time. Peter Williams only appears visibly in three scenes – in an interview with Lesley Brown’s Bristol gynaecologist, Dr Hinton, in a scene about halfway through the programme in which he explains early cell division in the embryo and in the operating theatre, when Patrick Steptoe shows him how to look down the laparoscope, which I will discuss later in this article.

## 2 Science on the Tube: Science Communication, Visual Culture and IVF

An extensive review of the literature on science communication, the relationship between visual culture and IVF and representations of science on television is well beyond the scope of this article, but I do want to draw out some crucial points in relation to the philosophy of science communication and the qualities of visual narrative as they relate to this particular story. Most importantly, I want to draw attention to the importance of normativity – both in form and content – in how this origin story was told in the popular media.

Not unusually, little of the media coverage of the birth of Louise Brown was by science or health correspondents, and Peter Williams does not have a science background either, though he did have a track record of covering science stories alongside current affairs when he started filming ‘To Mrs Brown’ in 1978. Nonetheless, there are some overlaps between the aims of scientists and journalists that are highly relevant to this case. In particular, scholars have noted that scientists and journalists both see themselves as seeking and representing truth, which can lead to contests for authority and authorship. In their study of media representations of human cloning, Joan Haran and colleagues note that such contests matter because they are based on an assumption that there is a fixed, objective and observable reality which can be gauged and described through science and/or journalism.^10^

Contests for truth between scientists and journalists came to the fore in the second half of the twentieth century. In her study of the first human-to-human heart transplant by South African surgeon Christiaan Barnard a decade before Louise Brown was born, Ayesha Nathoo discusses how the media portrayal of the event led to a shift in focus in science reporting from using television as a tool for teaching the public about science and medicine to a means for publicly debating the ethics and merits of particular techniques. The BBC programme that she discusses, ‘Barnard Faces His Critics’, was unprecedented in subjecting a medical researcher to questioning from diverse laypeople on the technical, social and ethical aspects of heart transplantation. So, by 1978, this kind of scrutiny of medical research had become more commonplace, yet in many ways ‘To Mrs Brown’, despite discussing the social and ethical implications of IVF as well as its technical specificities, was a largely didactic exercise – or, as Nancy Banks-Smith put it, a ‘nice, clear, uncritical job of work’ – in which Steptoe and Edwards were presented as normal, well intentioned and willing to answer questions rather than particularly needing to give an account of themselves or to justify their work.^11^

As Roger Silverstone argued, narrative (whether visual or linguistic) is crucial in the making and reception of television. He asserted that the language of television imposes a particular structure on the narrative it can produce; in other words, television is a typically conventional medium rather than an especially creative or innovative one. Similarly, while Silverstone noted that the relationship between television and ‘common sense’ is complex, he said that, ‘Like common sense … television translates history, political and social change, into manageable terms. … Television and common sense do not enquire into why things should be as they are, only that they are so but have somehow to be lived with. ‘Ron Curtis’ analysis of narrative form and normative force in science communication further adds to this case. As he argues, building a narrative (whether in science communication or any form of media representation) is neither a politically neutral nor an innocent process. In order to translate or tell a story about science – just as anything else – journalists, filmmakers and scientists create a narrative, but in doing so they select what they see as the pertinent points, the most suitable narrative form, main protagonists and so on; this editing work is itself normative. Curtis says,

Popular science, written in a narrative mode, is a powerful tool for promoting a particular normative view of science while, at the same time, rendering that view immune to criticism. It is a way to moralize while appearing only to describe. This is why the narrative mode is almost universal in popular science.^12^

As I will discuss, Peter Williams, like Steptoe and Edwards, subscribes to a traditional public-understanding-of-science (PUS) model of science communication. This model assumes that knowledge is a quantifiable thing that people do or do not have and that in order to understand research and innovation, the public simply needs to have its knowledge topped up by responsible scientists. As Haran and colleagues point out in relation to the more recent case of debates about human cloning, the PUS model reproduces a view of science as being led by individual scientist-pioneers, who are also the pivotal figures in science communication. While PUS rests on the assumptions that information is quantifiable and that scientific knowledge is objective, scientists and journalists use more ‘subjective’ narrative techniques in order to communicate their work. In her classic study of science journalism, Dorothy Nelkin described how scientists use metaphor to explain and popularise complex, technical and unfamiliar concepts. She wrote that many science journalists see themselves as politically neutral, based once again on the assumption that science is about objective facts. But this is a fallacy based on a limited and idealised view of both politics and science. As she said, tropes are not only explanatory aids but also strategic tools – in choosing particular words, symbols, analogies and images, scientists and journalists frame knowledge and its meaning.^13^

Television tells stories through a powerful juxtaposition of audio and visual information, which seems to bring journalistic and scientific stories to life. When it comes to communicating novel technologies to the public, seeing is thought to provide clarity, focus and the illumination of mysteries. One pertinent example of this is the way that British politicians described visiting infertility clinics in the 1980s during the parliamentary debates that led to the establishment of the Human Fertilisation and Embryology Act to ‘see for themselves’ what IVF entailed, and how this acted as a powerful counterexample to popular images of untrustworthy ‘mad scientists’ doing unthinkable things in secret labs. This exemplifies a culturally dominant faith in vision and a belief that sight is the primary sense – that the eye is synecdochic of the mind or even the whole person.^14^

ART relied on imaging technologies for their development,^15^ and Edwards and Steptoe’s account of their work on IVF, *A Matter of Life*, is strewn with references to the aesthetics of conception and the importance of visualisation to their progress. Laparoscopy is no longer standard practice in IVF, but it was vital in Steptoe and Edwards’ work as it was the best way to visualise the ovaries and thereby retrieve eggs for fertilisation in vitro at the time. Microscopy is another essential visual technology in IVF, allowing clinicians to visualise fertilisation and monitor developing embryos. As I will show, both of these technologies also played an important role in ‘To Mrs Brown’, providing viewers with an indication of what IVF looked like. Beyond the IVF clinic, there were many more intersections between visual technologies and reproductive politics in the latter decades of the twentieth century, from second-wave feminist self-help groups using specula and mirrors to visualise their genitalia, to the use of foetal imagery in struggles over abortion rights, to the adoption of the fourteen-day limit on embryological research based on the visible emergence of the ‘primitive streak’ in early embryos. These were not only an important part of the context in which IVF developed but also reflected a new questioning of medical and scientific authority, especially in the field of sexual and reproductive health.^16^

In IVF, seeing is important in a range of ways, including technical guidance and technological assistance in the clinic or laboratory, showing sceptics a different perspective, cultivating empathy with infertile people by seeing their point of view, encouraging faith in researchers by exposing their practices to scrutiny and enabling surveillance and oversight of reproductive scientists. One final visual technology that assisted in the development of IVF was journalistic photography and filmmaking. As noted, the birth of Louise Brown provoked a media maelstrom, and newspapers vied for photos of Louise and interviews with her parents. Steptoe and Edwards were also under pressure from newspapers to allow scrutiny of their work, so, as well as signing a syndication deal with Associated Newspapers, they and the Browns agreed to let the government’s Central Office of Information film the birth, which was then edited and shown on national television. Finally, it has recently been revealed that one of the major funders of the early research into IVF at Oldham was an American television executive, Lillian Lincoln Howell, though unfortunately it is not known why she decided to support the endeavour so generously.^17^

## 3 Looking into the Test Tube: Visual Verification of a Normal Baby

**Figure 2:**
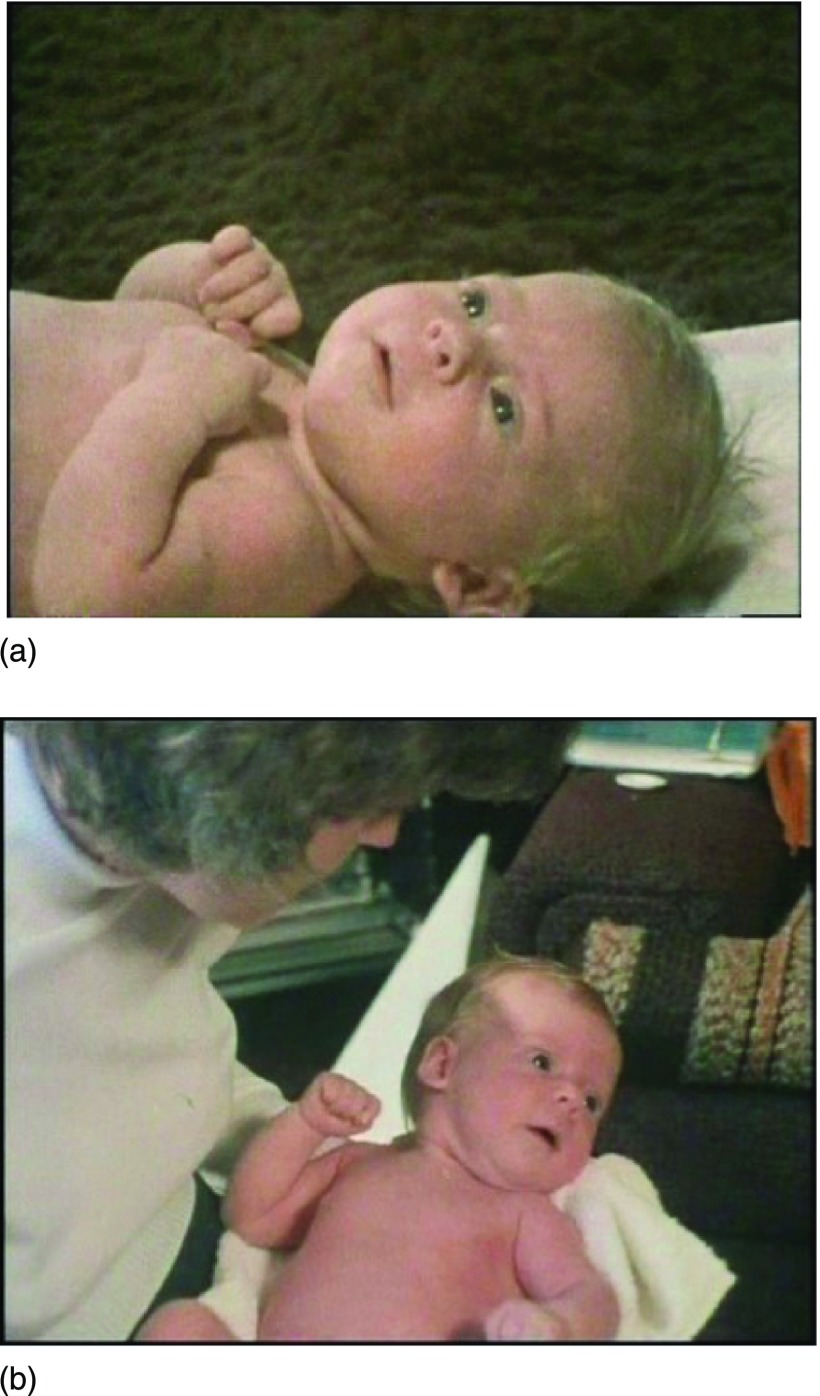
Louise Brown: ‘A normal child’, in ‘To Mrs Brown… A Daughter’.

As might be expected, one of the leading characters in the film is the daughter of its title, Louise Brown. The camera regularly returns to her throughout the programme, sometimes on her own and sometimes with her parents (Figure 2). In a scene ten minutes into the film, we observe a home visit from the health visitor. As Peter Williams’ voiceover assures us, this is just a routine check that any baby would have. In the scene, the health visitor, who wears a floral dress and pearls and has quite a ‘posh’ accent, weighs the baby and makes a comical intake of breath at her robust weight of 7lb 9oz, while John jokes in the background that they should ‘cut out the egg and bacon’ in her diet. The shot returns to Louise, now in her mother’s arms and staring curiously at the health visitor while she murmurs praise. This scene establishes that Louise Brown is a normal, healthy baby. In quite a few shots of Louise in the programme, she is naked. This gives a sense of her universality as well as suggesting that there is nothing that her parents or Steptoe and Edwards might wish to hide – she has no ‘abnormalities’, as they said at the time, a concern which was not only about the uncertainties of in vitro conception but which would have had an extra resonance as this was only a generation after the thalidomide disaster came to light in 1962.

This cheerful domestic scene is followed by a rather jarring one of laboratory experimentation on mice while Williams’ voiceover discusses the Medical Research Council’s decision not to fund Steptoe and Edwards’ work until they had done more trials on other species. Williams told me that he thought it important to show both sides of a debate in a documentary, and much of the second half of the programme is devoted to airing popular ethical concerns about IVF, though only through questions that he puts to Steptoe and Edwards – no one who actually objects to IVF is interviewed for the programme. These scenes of experimentation on mice, which are reprised in the second half of the programme when the voiceover discusses developmental biology research on rodents, symbolises the view of those who oppose IVF. Journalists typically strive for ‘balance’ in their work, which rests on the assumption that there are two sides to any story, and public debates about the ethics of IVF and ART have been characterised by such binarism throughout their history.

While, as noted, in 1978, the British press presented a largely positive picture of IVF as a means of helping infertile couples to have children, by the 1980s, there was a growing sense, which was evident in both media and parliamentary debates, that IVF and other ART were not ethically straightforward. Typically, the debate was depicted as a two-sided one: on the one hand, the ‘pro’-IVF camp represented itself as sympathetic to infertile couples and positively inclined towards scientific ‘progress’; on the other hand, the ‘anti’-IVF camp was primarily morally concerned about scientists overreaching or ‘playing god’. While some journalists and proponents of IVF assumed or implied that anti-sentiment was driven by religious dogma, this was not necessarily the case, as some who were concerned about IVF felt that the technology was problematic not because it was contrary to specific religious teachings but because it appeared to be tinkering with the natural order or the institution of the family. There were also robust debates within feminism, though these were much less represented in the media or parliamentary debates, about whether these technologies were liberatory or repressive. The scenes of mice in laboratories represent the ‘balance’ to the sight of a normal, healthy, blonde, blue-eyed baby. It seems that Peter Williams trusted the viewers, armed with what they would have learnt from watching the programme, to weigh these images against each other and decide that experimentation on mice (which, although it is common laboratory practice, is still somewhat opaque to most lay members of the public) was of little concern compared to the happiness brought by a healthy human baby.^18^

In the final scenes of the programme, Williams interviews Lesley, who is shown in close-up, and asks her whether she is worried about Louise living with the label of ‘test-tube baby’ as she grows up. Lesley replies that she hopes with time the attention will lessen so that Louise can lead a normal life. Williams suggests, ‘A normal life, for a normal child?’ Lesley smiles slightly and says, ‘She is, isn’t she?’ The following, final scene returns to the shot of Louise lying on her back, waving her arms in the air, as the credits roll.

## 4 ‘Knowledge is Power’ and Seeing is Believing: Peter Williams’ Role in the Programme and His Aim in Making It

In the programme, Peter Williams – a white, middle-aged man with, as noted, a resonant Received Pronunciation accent – provides an authoritative guiding voice and acts as a mediator between the scientist and the viewer, though, as we shall see, his identity within the film is at times rather closer to Patrick Steptoe’s than the viewer’s. Williams described to me in an interview how he had been motivated to make the film. As well as admitting that it appealed for its potential as a scoop, he had much sympathy for the cause of treating the ‘agony’ of infertility and real respect for Steptoe and Edwards. He had already met Steptoe socially in Canterbury, near where he lives and where Steptoe had a holiday home, and had first filmed him performing laparoscopy in 1975. In our interview, Williams made it clear that he saw his role in making this film as showing the public that IVF was safe and morally acceptable.^19^

I asked Williams how the programme had come about and he explained:

… at that time I was working for Thames [Television] and I just thought it was a good idea. … Roughly about that time, I made the very first documentary about what it was like to be an astronaut. So I went to the [United] States and … well, Buzz Aldrin was among the people we talked to and again you have to get through barriers. You have to explain to people why it’s important that the world outside knows what they’re doing. And that applied as much to men going to the Moon as it did to men trying to create, or teams trying to create, the first IVF baby. It’s really important to carry a public opinion along with them. And I’m sure Bob [Edwards] and Patrick [Steptoe] recognised that, and they simply had to decide who they wanted to do it. And so I suppose I was there early on and I was consistently interested and rather like an old piece of furniture, [so] they decided I should do it.

Williams mentioned the comparison between the first landing on the Moon and the first IVF baby a couple of times in our interview. Williams had heard Steptoe remark to a colleague just before Louise’s birth, ‘You do realise, don’t you, that this story is bigger than man landing on the Moon?’ This analogy points to the significance of the birth in historic terms, its journalistic appeal and a sense of what it meant for both Steptoe’s and Williams’ careers. It also points to the importance of marking firsts in both journalism and science, and Steptoe’s awareness of this. Another interesting parallel that emerges from Williams’ comments above is that he was not just interested in reporting world firsts in these two documentaries but in getting across what it is like to be involved in them, thereby helping viewers sympathise with the protagonist(s).^20^

Williams told me that he had had a sense that IVF was morally right from the start and that he had been sceptical of the risks that some posited for the technique before 1978. He recalled some ‘vigorous dinner party conversations’ with friends and peers who were worried about the implications and consequences of the technology. His answer to this, he said, had been to follow Steptoe’s line, pointing out that infertility ‘is as much an illness as anything else’, so ‘we must do what we can to try to alleviate it’. He said that once he had spelt this out, it usually led to the ‘beginning of an understanding’. With ‘To Mrs Brown’, he was attempting to have the same effect on a larger scale and through a visual as well as verbal medium.

In his account of how the film came to be made, Williams talked about both convincing the protagonists of the need to share their stories with the outside world and of the importance of carrying public opinion along with them. Steptoe and Edwards needed little persuasion on this point. They were unusually willing to discuss their work with the media and they understood the need for popular approval for it to continue. It seems that they chose Williams for the job because Steptoe already knew and trusted him but also because of his pre-existing sympathy for IVF. In particular, he seems to have shared with Steptoe and Edwards a view that if scientists are open to public scrutiny and can give a good account of what they are doing, then they will be able to secure public assent. This is in line with the PUS model mentioned earlier, which assumes that the public will be persuaded of the acceptability of a new technology if they are given enough information to understand both the technique and its applications.^21^

**Figure 3:**
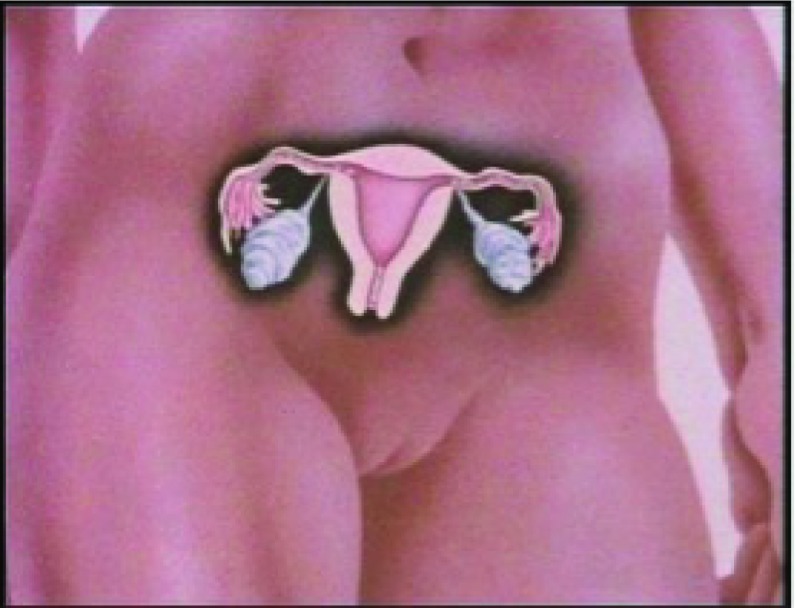
Diagram of the female reproductive system from ‘To Mrs Brown... A Daughter’.

In ‘To Mrs Brown’, IVF is explained in various ways and registers. The interviews with Lesley and John Brown tell how they overcame their infertility and found happiness through being treated by Patrick Steptoe; the interviews with Steptoe and Edwards give an account of how these two pioneers came to work together and solve the puzzle of how to make extra-corporeal conception work; the interviews with other infertile couples make the viewer aware of what infertility means and the hope that IVF represents to the broader public; and the clinical scenes provide a visual narrative of how IVF is done. Along with this, Williams and his team included some diagrams of the female reproductive system and the process of conception, which they spliced with footage of sperm under the microscope and stills of embryos and foetuses. They drew these animations themselves, in consultation with Edwards and Steptoe. They are reminiscent of the images many viewers would have seen in educational videos in biology class at school (Figure 3).

In discussing the animations in our interview, Williams articulated the idea behind them as well as his broader sense of how visual communication might carry the public along with Steptoe and Edwards’ work:

we were talking to an audience who were both apprehensive about what was going on but fascinated at the same time. And, you know, they say knowledge is power; knowledge also leads to understanding and calmness and, you know, it gives … both sides of the story if there are two sides to it. And I think we were simply doing a plain… in those days it was called a ‘plain man’s guide’ to IVF, really.

**Figure 4:**
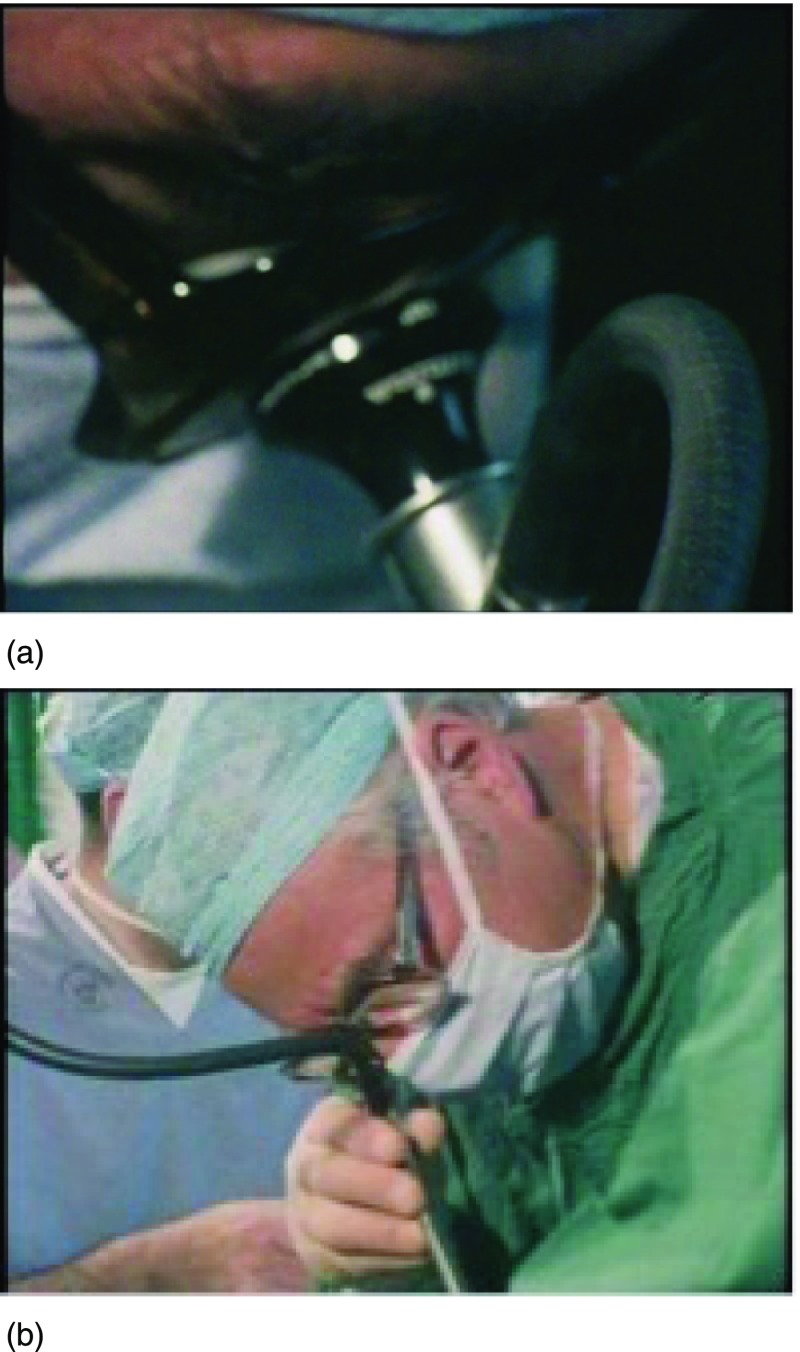
Peter Williams (a) and Patrick Steptoe (b) look into the laparoscope in ‘To Mrs Brown… A Daughter’.

## 5 The Biological Gaze and the Journalistic Lens

Peter Williams is clear that trust is crucial in documentary making, as it facilitates access and establishes sympathy. This is true of the relationship between viewer and programme-maker but also between programme-makers and the protagonists of their films. In ‘To Mrs Brown’, Williams acts not only as mediator between the protagonists and the viewer but also as a student and occasional double of Steptoe. This is exemplified by a scene of visual pedagogy seven minutes into the film, when Steptoe shows him how to look down the laparoscope. This is also the first scene (of only three) in which Williams is – albeit briefly – visible in the programme. Figure 4 juxtaposes two screenshots: on the bottom, Patrick Steptoe looks down his laparoscope in the theatre; on the top is a near mirror image but with Williams looking down the same laparoscope under Steptoe’s audible guidance. Both are wearing surgical scrubs, masks and caps; both wear thick dark-rimmed glasses. The areas around their eyes, one of the few parts of their bodies that are visible, are in the centre of the screen. The viewer’s gaze is drawn to the gaze of the man looking down the laparoscope and, as if to satisfy her curiosity, the scene is cut with laparoscopic views inside the body. The scene in which Steptoe guides Williams to see what he sees illuminates the biological mysteries to which Steptoe is privy; it shows that Steptoe is not secretive about his work and demonstrates that he is eager to teach others.

**Figure 5:**
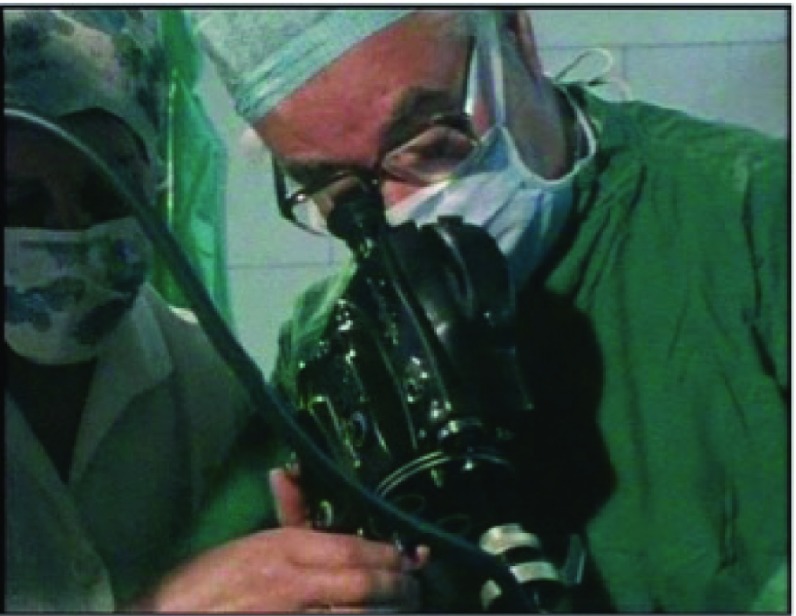
Steptoe films his laparoscopy in ‘To Mrs Brown… A Daughter’.

Another notable feature of the extensive clinical footage in ‘To Mrs Brown’ is a somewhat hall-of-mirrors scene in which Williams and his crew film Steptoe and his surgical team filming a laparoscopy using a Beaulieu R16 camera (Figure 5). This scene comes nearly twenty minutes into the programme and it leads up to the ‘reveal’ of what IVF actually looks like. Over the course of six minutes, the film shows the clinical procedure of IVF and the partnership between Steptoe and Edwards in action. The fact that Steptoe decided to narrate much of this section as if he were giving a tutorial as well as filming the laparoscopy indicates his confidence, expertise and flair. It implies that IVF is so straightforward (for him, at least) that it can be combined with other tasks and that it is almost routine in his practice.^22^

During the scene, Steptoe is seen performing egg retrieval by laparoscopy. The woman on whom the laparoscopy is being performed is unnamed, but in his voiceover Williams describes Lesley Brown’s experience, so in the programme’s narrative she represents Lesley. Edwards is in the adjoining laboratory and Steptoe talks to him via an intercom as he performs the egg retrieval, which they refer to as the ‘aspiration’. Having set up the laparoscopy, Steptoe starts describing the equipment, presumably to Williams, though the effect is as if he is talking directly to the viewer. The camera follows his lead, focusing where he indicates. Then, Steptoe starts to film the laparoscopy. The theatre sister holds the 16 mm camera to the eyepiece of the laparoscope while Steptoe looks into the camera and guides the sister in using it, as if it were just another surgical instrument. The film then cuts to surgical footage, implicitly that taken by the Beaulieu R16 at the time, replicating for the viewer what Steptoe sees himself. The number of lenses that mediate this view, as illustrated in the screenshot in Figure 5, is striking: Steptoe’s eye, his glasses, the camera and finally the laparoscope. The laparoscopy footage is cut with footage of the collecting chamber as the follicle is aspirated, following the bodily fluids that are being extracted on their journey from in vivo to in vitro. At this point, Steptoe declares to Edwards over the intercom that the aspiration is ‘excellent so far’. A member of theatre staff then takes the small collecting chamber through to Edwards’ assistant Jean Purdy in the laboratory; Purdy places it on the viewing stage of the microscope at which Edwards is seated.

**Figure 6:**
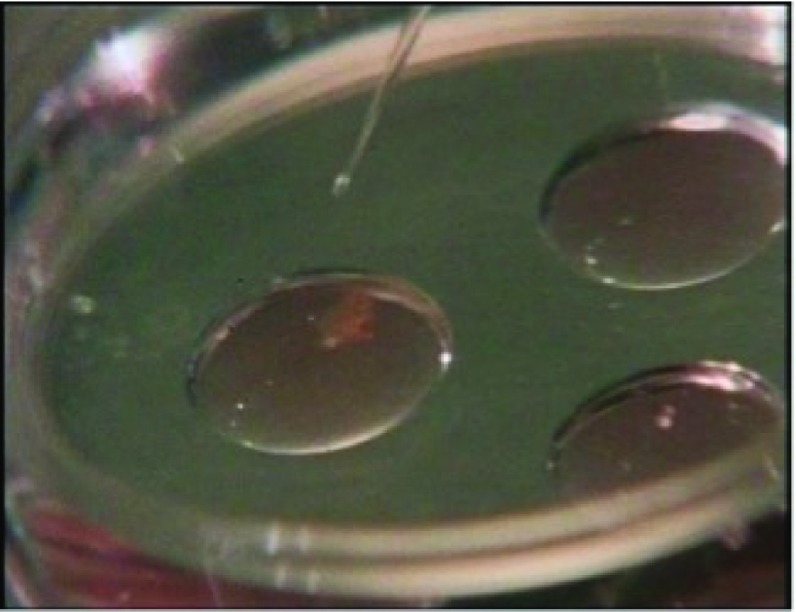
‘In she goes’ – the act of in vitro fertilisation as shown in ‘To Mrs Brown… A Daughter’.

The scene now switches to Robert Edwards’ work in the laboratory. He is shown examining the contents of the collecting chamber under the microscope, removing the egg and placing it in culture medium in a petri dish. Like Steptoe, he narrates his actions, along with some voiceover additions by Williams. While Steptoe had a rather patrician manner and resounding Received Pronunciation accent, Edwards’ voice had a soothing tone and he retained his Yorkshire accent despite his travels in the UK and abroad. This probably made him a less intimidating character than Steptoe, which could have helped his laboratory surroundings seem less daunting to many viewers.^23^ Edwards describes placing the egg in the culture medium as ‘putting it home’ and declares that ‘now the egg is safe’. He looks at it again under the microscope and announces it is ‘a lovely egg, a very nice egg indeed’. Edwards explains that he has ‘the husband’s sperm’ in another dish and checks them under the microscope, affirming that it is ‘an excellent preparation’. He then ‘introduces’ the egg to the sperm. The egg is in a pipette and an extreme close-up shows him placing the egg with the sperm in the dish (Figure 6). The reversal of gender roles from standard biological descriptions of fertilisation is not remarked upon, though Edwards does announce, ‘in she goes’ as he releases the egg from the pipette and then comments, ‘beautiful’. Peter Williams then declares in the voiceover: ‘this is the act of in vitro fertilisation; the moment of conception outside the human body’.

This section, then, is Williams’ ‘plain man’s guide to IVF’. It is critical because it would have been the first time the vast majority of its viewers had seen the moment of fertilisation at all, let alone in a petri dish. This scene was Williams’ Man on the Moon moment, yet – like Louise Brown herself – despite its miraculous nature it appears quite ordinary. While the setting of the laboratory might have been formidable to many television viewers, the quiet concentration and warmth of Edwards, along with his unscientific and romantic language of home, beauty and safety as he is performing the fertilisation, make IVF medical and domestic rather than monstrous.

Once again, this scene, showing the journey from egg retrieval to fertilisation, depicts Steptoe and Edwards as completely open about their work, happy not only to have it filmed but also to try and explain it to the viewer. This reflects the common goals of Williams, Steptoe and Edwards of explaining IVF – both verbally and visually – in order to carry public opinion along with them. The fact that Steptoe already filmed his own work and that he and Edwards both look comfortable explaining it on camera underlines that these three men not only had similar aims but also common approaches to achieving them.

In the case of the first IVF baby, Steptoe and Edwards were able to guide the public to see IVF in a particular way – in the manner that they explained IVF verbally, through forming relationships with sympathetic journalists (and excluding others, as exemplified by the syndication deal that they brokered between the Browns and Associated Newspapers) and by directing how IVF was visually represented. The scene in ‘To Mrs Brown’ in which Steptoe guides Williams’ vision down the laparoscope is one example of this, demonstrating that the scientist can show the journalist how and what to see. Here there is also a kind of telescoping at play, as Steptoe is not only showing Williams how to see IVF, so that he knows what to show the viewer of his programme, but also simultaneously usurping Williams by talking to the viewer directly himself. This is the hall-of-mirrors effect: not content with taking over direction and presentation of the film on the hoof, Steptoe shows the filmmaker how to film laparoscopy; having just invited Williams to mimic his laparoscopic gaze, Steptoe goes on to imperfectly reproduce Williams’ role as filmmaker. That Williams has accepted this vision of reality is affirmed by his inclusion of this scene, including the laparoscopic footage, in his film and by his placing it at a pivotal point in the programme’s narrative. This is reinforced by the fact that there is a small audio glitch in the scene, which an experienced filmmaker like Peter Williams would probably rather have edited out unless he judged it absolutely necessary to include it.

In her essay on the biological gaze, Evelyn Fox Keller discusses the ways in which looking is tied up with touching in embryological science. This is not merely a question of moving objects around or extracting specimens in order to view them but of the changes to the subject being observed that that necessarily entails. As she puts it, ‘what we see as we gaze at the secret of life is life already, and necessarily, transformed by the very technology of our gaze’. Keller points out that this is significant not only for those things being manipulated and gazed at in the exercise but also for the viewer, because such actions contribute to a sense of what is real. The biological gaze, like the journalistic or documentary one, is aimed at seeing, and showing, reality. Having control over how embryos, and embryology, are seen is therefore a matter of power – the power to represent reality and thereby claim access to truth.^24^

## 6 Discussion: The Politics of IVF on Screen

Peter Williams, Patrick Steptoe and Robert Edwards believed that IVF was a moral and helpful technology that would reduce suffering and bring happiness. ‘To Mrs Brown’ enabled these three men to frame what IVF looked like and what it meant at a pivotal point in its history. The frequent shots of Louise Brown at six weeks old with her unblemished skin, soft blonde hair, big blue eyes, button nose and chubby limbs are those of a quintessentially bonny baby. Like the snapshots of smiling newborns that adorn the walls of fertility clinics today, these shots denote the hope for the infertile that IVF has come to symbolise ever since she was born. Babies signify happiness and the future, and by representing baby Louise as the outcome of IVF, the technology became associated with both. Of course, not all futures are happy, but rather than the kind of anxious futures expressed in science-fiction representations of assisted reproduction or the public fears that emerged in the embryology debates during the 1980s, IVF in 1978 was associated with the happy, wholesome future that is popularly epitomised by children and family. IVF was represented as a potential solution to the negative pregnancy tests, miscarriages, depression, marital strain and social stigma associated with infertility rather than a technology that opened up opportunities for scientists to create life according to their own designs or prejudices, or for people to assume control over what had previously been thought of as the dominion of God or Nature.^25^

Steptoe, Edwards and Williams shared a faith in the idea that if the public are informed about scientific innovations, they will understand and come to accept them calmly. ‘To Mrs Brown’ added a further visual dimension to the story of the birth of Louise Brown in the newspaper articles (and their accompanying black-and-white still photographs). Visual communication is especially powerful: it is popularly thought of as more capable of capturing reality and less open to manipulation than verbal communication, though of course it is highly malleable. In ‘To Mrs Brown’, Williams represented Steptoe and Edwards as very willing to discuss their work on IVF, its applications and its ethical implications. They appear skilled, knowledgeable, amenable to discussion and driven to help others. Alongside these verbal assurances, cheerful images of Louise Brown, the perfectly normal baby, her loving, ordinary parents and the quiet scene of ‘the moment of in vitro fertilisation’ showed viewers that the men who helped make her were not faceless Frankensteins but people with personalities, families and particular tastes in home furnishings.^26^

Peter Williams seems to be well aware of the political questions surrounding IVF, and he mentioned politics a couple of times during our interview without prompting from me. He noted that Steptoe and Edwards as individuals had diametrically opposed political views – he described Steptoe as ‘a High Tory who wore cravats and played the grand piano’ and recalled that Edwards was a socialist Labour Party councillor in Cambridge – and wondered aloud if they had ever talked about politics. In the film, he devotes some attention to the fact that all of Steptoe’s patients had signed a form consenting to undergo a termination of their pregnancy if amniocentesis revealed abnormalities. We discussed this in the interview and he said, ‘I do think that [Steptoe and Edwards] felt that politically they couldn’t afford to have a child, the first child, born deformed. So they wanted to count the fingers, and they wanted to count the toes, and they wanted to see that there weren’t two heads.’ As this suggests, if Louise had been born anything other than visibly ‘perfect’, it could have finished the whole project.

Williams also mentioned that Edwards had worked in the United States earlier in his career and so was well aware of the strong influence of the ‘pro-life’ lobby on American politics, suggesting that this had made him and Steptoe cautious about presenting their work to the public. This would likely have been reinforced by the criticism they had received when news of their experimentation had first emerged a decade earlier. In the late 1960s and early 1970s, the public reaction was far more critical than it was by the time of Louise Brown’s birth. Edwards and Steptoe’s account of several examples of this in *A Matter of Life* shows that they were stung by this criticism, especially when it came from their peers in the scientific world, as in the example of James Watson’s florid dismissal of their work on IVF in an American panel discussion in 1971.^27^

In our interview, Williams did not acknowledge that IVF is political in another way – its potential for normativity. In 1978, IVF was envisaged as being primarily aimed at helping married heterosexual infertile couples have a baby, and Steptoe was very clear that he believed that this should be its exclusive purpose. This was the specific kind of happiness and hope that it represented at the time. Proponents of IVF in the early days often draw attention to the criticism and resistance that Steptoe and Edwards met from the media, politicians and theologians. Williams also implied this in our interview when he described what it was like to be at the eye of the media storm surrounding Louise Brown’s birth. It is certainly true that this criticism existed, but I have read and analysed the newspaper coverage from 1978 and while journalists often described IVF as ‘controversial’, it was incredibly rare for them to actually give a platform to critics of IVF at this time. The dominant narrative about IVF in the British media in 1978 was supportive and celebratory rather than critical of Steptoe and Edwards’ work.^28^

Roger Silverstone argued that, ‘It is in making the unacceptable acceptable, in clarifying ambiguity and strengthening resistance to uncertainty that the television narrative gains its significance.’ Peter Williams was motivated, out of sympathy both to Steptoe and Edwards and to those who suffered infertility, to show that IVF was morally acceptable. He tried to do so, in line with the traditional PUS model, by giving viewers of his programme more information about IVF, through the narrative of the Brown family’s new arrival, Steptoe and Edwards’ explanations of their research that led to Louise Brown’s conception, interviews with infertile couples that focused on the sadness and disappointment their condition had brought them and a chance to see what IVF looked like.^29^

In contrast to the PUS model, H.M. Collins argues that it is not more information that would help improve people’s understanding of science but ‘reflective understanding’. Collins notes that members of the public are typically asked to decide on scientific issues that are controversial when there is disagreement between experts, and that they will do so based not just on their understanding of the science but on more ‘everyday criteria’, such as who appears more plausible or trustworthy. It behoves scientists, therefore, to be savvy about appealing to the public, just as Steptoe and Edwards recognised in their own engagements with the British media. The reflective understanding that Collins refers to is ‘the way science seems to generate certainty and what this means for its relationship to other kinds of knowledge’. Programme-makers like Peter Williams are important in creating certainty, and for Collins, this is especially so with controversial topics, which is when journalists are more likely to offer their own opinions. Collins asserts that when journalists and filmmakers intervene in this way, viewers and readers are more likely to take the programme-makers’ opinions as decisive. As this case suggests, this is all the more powerful when there is accord between the filmmaker and the scientists whom they are representing.^30^

By emphasising the criticism that ART has accrued over the years, supporters of Steptoe and Edwards like Peter Williams obscure the power that they have had to represent IVF and thereby contribute to its normalisation. In the embryology debates that followed Louise’s birth in the 1980s, a narrative eventually emerged in which IVF was associated with progress and the noble cause of helping the infertile and carriers of genetic diseases, while those who were against it were often painted as anti-science. This implied that resistance to IVF was irrational, caused by a lack of information or an inability to understand it. This caricature concealed the point that what was being contested was not what the science of IVF is, which is relatively straightforward, but what IVF is for, what it means and what it could lead to. Focusing on the ‘facts’ of IVF and providing a ‘plain man’s guide’ obscures the political, ethical and aesthetic decisions entailed in representing IVF – and the normative and political effects of such decisions on both the availability and the meanings of such technologies.^31^

## 7 Conclusion

For Roger Silverstone, television is ‘a political entity, but a political entity with a small “p”, that is fully implicated in the interweaving of both the small-scale and the large-scale manifestations of ideology and control that provide the framework of life in modern society’. Technologies like television are subtly yet powerfully political because of their association with the everyday and domestic realm, both of which are popularly thought of as apolitical, yet which play a crucial role in the formation and reproduction of norms. IVF is not yet an everyday technology, but it has become routinised and, in the UK at least, largely normalised. This normalisation has occurred not only because of the technology’s usefulness as a platform for other technologies but also because of its close association with the hope and happiness brought by much wanted babies like Louise Brown since 1978.^32^

In order to understand the politics of IVF it is necessary to place it in its historical context alongside other forms of reproduction, which includes recognising the political and normative ideas that have informed its representation. Scientists and journalists are both interested in discovering and representing truth. For both, authority results from proximity to truth. Peter Williams, like Robert Edwards and Patrick Steptoe, believed the true story of IVF was one of hope for infertile couples who wanted to have children of their own. All three men also recognised that in order to secure support for their work on IVF from the public, patients, funders, government and their peers they needed to inform them that this, and not the dystopian visions of Mary Shelley or Aldous Huxley, was the true story of IVF. The three men agreed both that their goal was to ‘carry public opinion along with them’ and that the means to do so was to harness media representations of IVF to this end. Through a potent combination of scientific authority, editorial certainty, everyday realities and visual truths, ‘To Mrs Brown… A Daughter’ played an important part in establishing the dominant narrative of the origin story of IVF, thereby contributing to its normalisation.

## Footnotes

1.
Peter Williams, personal interview, London, 17 March 2015; Peter Williams, Screening of ‘To Mrs Brown… A Daughter’ with Q&A. IVF Histories and Cultures Project (IVFHCP) Workshop 2, Christ’s College, University of Cambridge, 11 December 2014.

2.
Stuart Hall, ‘Representation and the Media’ (Media Education Foundation transcript, 1997), http://www.mediaed.org/transcripts/Stuart-Hall-Representation-and-the-Media-Transcript.pdf, accessed 3 October 2014.

3.
‘Son of This Week’, *Daily Mail*, 2 September 1978, 20; Tom Buckley, “‘Test-Tube Baby” Documentary’, *New York Times*, 4 February 1979; Nancy Banks-Smith, ‘TV Eye’, *The Guardian*, 8 September 1978, 8.

4.
Lisa H. Harris, ‘Challenging Conception: A Clinical and Cultural History of In Vitro Fertilization in the United States’ (unpublished PhD thesis: University of Michigan, 2006); Robin Marantz Henig, *Pandora’s Baby: How the First Test Tube Baby Sparked the Reproductive Revolution* (Cold Spring Harbor, NY: Cold Spring Harbor Laboratory Press, 2004); see Katharine Dow, “‘Now She’s Just an Ordinary Baby”: The Birth of IVF in the British Press’, *Sociology* (published online first: 22 February 2018) and “‘The Men who Made the Breakthrough”: How the British Press Represented Patrick Steptoe and Robert Edwards in 1978’, *Reproductive Biomedicine and Society Online*, 4 (2017), 59–67; M.H. Johnson *et al.*, ‘Why the Medical Research Council Refused Robert Edwards and Patrick Steptoe Support for Research on Human Conception in 1971’, *Human Reproduction*, 25, 1 (2010), 2157–74; Sarah Franklin, ‘Deconstructing “desperateness”: the social construction of infertility in popular representations of new reproductive technologies’, in Maureen McNeil, Ian Varcoe and Steven Yearley (eds), *The New Reproductive Technologies* (London: Macmillan, 1990), 200–29.

5.
It seems likely that the whiteness of the Brown family helped in garnering support for IVF, especially in the more conservative tabloid newspapers, not least because it helped them appear ‘ordinary’ in a predominantly white nation which was, in 1978, experiencing considerable racial tension. However, it is notable that of the four other infertile couples seeking treatment with Steptoe that appear in ‘To Mrs Brown’, two are white, one is mixed-race and one is black and their ethnicities are never remarked upon.

6.
Sarah Franklin, ‘Life Story: The Gene as Fetish Object on TV’, *Science as Culture*, 1, 3 (1998) 92–100: 99; see also Sarah Franklin, *Biological Relatives: IVF, Stem Cells, and the Future of Kinship* (Durham, NC: Duke University Press, 2013); Raymond Williams, *Television: Technology and Cultural Form* (London: Fontana, 1974); Stuart Hall, *op. cit.* (note 2); see José van Dyck, *Manufacturing Babies and Public Consent: Debating the New Reproductive Technologies* (New York: New York University Press, 1995).

7.
Peter Williams, *op. cit.* (note 1).

8.
Jon Turney, *Frankenstein’s Footsteps: Science, Genetics and Popular Culture* (London: Yale University Press, 1998).

9.
D. Morley and R. Silverstone, ‘Domestic Communication – Technologies and Meanings’, *Media, Culture & Society*, 12 (1990), 31–55; Roger Silverstone, *Television and Everyday Life*, (New York: Routledge, 1994), 24.

10.
J. Haran *et al.*, *Human Cloning in the Media: From Science Fiction* *to Science Practice* (London: Routledge, 2008), 124.

11.
See also Allan Jones, ‘Elite Science and the BBC: A 1950s Contest of Ownership’, *British Journal for the History of Science*, 47, 4 (2013), 701–23; see also Timothy Boon, *Films of Fact: A History of Science in Documentary Films and Television* (London: Wallflower Press, 2008); Ayesha Nathoo, *Hearts Exposed: Transplants and the Media in 1960s Britain* (London: Palgrave Macmillan, 2009), 186.

12.
Roger Silverstone, *The Message of Television: Myth and Narrative in Contemporary Culture* (London: Heinemann Educational Books, 1981), 1, 182; Ron Curtis, ‘Narrative Form and Normative Force: Baconian Story-Telling in Popular Science’, *Social Studies of Science*, 24, 3 (1994), 419–61, 424, 425, 434–5.

13.
See also B.V. Lewenstein, ‘Science and the media’, in S. Jasanoff *et al.* (eds), *Handbook of Science and Technology Studies* (Thousand Oaks, CA: Sage, 1995), 343–61; Haran *et al.*, *op. cit.* (note 10), 75; Dorothy Nelkin, *Selling Science: How the Press Covers Science and Technology* (New York: W.H. Freeman & Co., 1987), 10; see also Emily Martin, *The Woman in the Body: A Cultural Analysis of Reproduction* (Boston, MA: Beacon Press, 2001 [1987]).

14.
See Jesse Olszynko-Gryn and Patrick Ellis, “‘A Machine for Recreating Life”: An Introduction to Reproduction on Film’, *British Journal for the History of Science*, 50, 3 (2017), 383–409: 384 for a similar point about reproduction in the cinema; Michael Mulkay, *The Embryo Research Debate: Science and the Politics of Reproduction* (Cambridge: Cambridge University Press, 1997); Sarah Franklin, ‘Making representations: the parliamentary debate on the Human Fertilisation and Embryology Act’, in J. Edwards *et al.* (eds), *Technologies of Procreation: Kinship in the Age of Assisted Conception* (Manchester: Manchester University Press, 1993), 127–65.10.1017/S0007087417000632PMC596343728923123

15.
Indeed, it is worth remembering that *in vitro*translates as ‘in glass’, and that glass is used to make both petri dishes and the lenses used in visual technologies.

16.
Robert Edwards and Patrick Steptoe, *A Matter of Life: The Story of a Medical Breakthrough* (London: Sphere Books, 1980), 94–5; Michelle Murphy, *Seizing the Means of Reproduction: Entanglements of Feminism, Health, and Technoscience* (Durham, NC: Duke University Press, 2012); Rosalind Pollack Petchesky, ‘Fetal Images: The Power of Visual Culture in the Politics of Reproduction’, *Feminist Studies*, 13, 2 (1987), 263–92; Sarah Franklin, ‘Postmodern Procreation: Representing Reproductive Practice’, *Science as Culture*, 3, 4 (1993), 522–61.

17.
Louise Brown, *My Life as the World’s First Test-Tube Baby* (Bristol: Bristol Books, 2015); Martin H. Johnson and Kay Elder, ‘The Oldham Notebooks: An Analysis of the Development of IVF 1969–1978. VI. Sources of Support and Patterns of Expenditure’, *Reproductive Biomedicine and Society Online*, 1, 1 (2015), 58–70.10.1016/j.rbms.2015.04.006PMC534128628299365

18.
Michael Mulkay, *op. cit.* (note 14); Sarah Franklin, *op. cit.* (note 6).

19.
T. Boon, ‘Formal Conventions in British Science Television’, *Actes d’Història de la Ciència i de la Tècnica, Nova Epoca*, 7 (2014), 51–69.

20.
Evelyn Fox Keller also describes an historical association between embryology and astronomy; see Evelyn Fox Keller, ‘The biological gaze’, in J.B. Bird *et al.* (eds), *Futurenatural: Nature, Science, Culture* (London: Routledge, 1996), 107–21.

21.
Edwards and Steptoe, *op. cit.* (note 16).

22.
According to the records of the British Film Institute, Steptoe directed an educational film, *Operative Laparoscopy in Gynaecology*, in 1971 (http://www.bfi.org.uk/films-tv-people/4ce2b6cd7d784, accessed 25 July 2016). Steptoe’s interest in film is further suggested by the fact that before his medical career, he had worked playing piano as a cinema accompanist. Edwards and Steptoe *op. cit.* (note 16), 65.

23.
In a couple of instances during his interviews elsewhere in the programme, Edwards even does an affectionate impersonation of Steptoe, putting on a posh accent and speaking in a forthright tone.

24.
Evelyn Fox Keller, *op. cit.* (note 20), 120, 113.

25.
Jon Turney, *op. cit.* (note 8).

26.
See also Jesse Olszynko-Gryn, ‘Thin Blue Lines: Product Placement and the Drama of Pregnancy Testing in British Cinema and Television’, *British Journal for the History of Science*, 50, 3 (2017), 495–520.10.1017/S0007087417000619PMC596343628923127

27.
Robert Edwards and Patrick Steptoe, *op. cit.* (note 16), 125.

28.
Martin H. Johnson and Kay Elder, ‘The Oldham Notebooks: An Analysis of the Development of IVF 1969–1978. IV. Ethical Aspects, *Reproductive Biomedicine and Society Online*, 1, 1 (2015), 34–45; Mulkay, *op. cit.* (note 14); Edwards and Steptoe, *op. cit.* (note 16); Katharine Dow, *op. cit.* (note 4).10.1016/j.rbms.2015.04.002PMC534128928299363

29.
Roger Silverstone, *op. cit.* (note 12), 8.

30.
H. M. Collins, ‘Certainty and the Public Understanding of Science: Science on Television’, *Social Studies of Science*17 (1987), 689–71, 691, 692, 709.

31.
Michael Mulkay, *op. cit.* (note 14).

32.
Roger Silverstone, *op. cit.* (note 9), 52; Sarah Franklin, *op. cit.* (note 6) 2013.

